# Diagnosis and treatment of oral focal mucinosis: a case series

**DOI:** 10.1186/s13256-019-2033-8

**Published:** 2019-04-26

**Authors:** Yusuke Higuchi, Fumihiko Tsushima, Kanako Sumikura, Yuriko Sato, Hiroyuki Harada, Kou Kayamori, Tohru Ikeda

**Affiliations:** 1 Oral and Maxillofacial Surgery, Department of Oral Restitution, Division of Oral Health Sciences, Graduate School, Tokyo Medical and Dental University, 1-5-45 Yushima, Bunkyo-ku, Tokyo, 113-8510 Japan; 2 Oral Pathology, Department of Oral Restitution, Division of Oral Health Sciences, Graduate School, Tokyo Medical and Dental University, 1-5-45 Yushima, Bunkyo-ku, Tokyo, 113-8510 Japan

**Keywords:** Gingiva, Oral focal mucinosis, Retromolar region

## Abstract

**Background:**

Oral focal mucinosis, the oral counterpart of cutaneous focal mucinosis, is a rare disease. As it has no characteristic clinical or radiological features, diagnosis is established by histopathological and immunohistological examination. We present three cases of oral focal mucinosis occurring in the retromolar (which is extremely rare) and gingival regions.

**Case presentation:**

Case 1 involved a 26-year-old Japanese man with radiolucency in the right retromolar region on panoramic radiograph and computed tomography; no obvious protrusion was observed in the region. This finding was clinically diagnosed as a tumor of the retromolar region. Case 2 involved a 60-year-old Japanese woman. A tumor-like mass of tissue was identified on the buccal gingiva at the maxillary right canine and first premolar region. The lesion measured 7 × 6 mm and exhibited elastic hardness and healthy-colored mucosa. The lesion was diagnosed as an epulis. Case 3 involved a 47-year-old Japanese woman. A tumor-like mass of tissue was identified on the buccal gingiva at the maxillary right canine and first premolar region. The lesion measured 10 × 10 mm and exhibited elastic hardness and redness of the surface mucosa. This lesion was also diagnosed as an epulis. Resection was performed in all three cases, and the lesions were histopathologically diagnosed as oral focal mucinosis. Postoperative courses were uneventful and, thus far, there have been no recurrences.

**Conclusions:**

Although it is difficult to diagnose oral focal mucinosis based on clinical symptoms and imaging findings, the disease should be considered a possibility when diagnosing benign oral tumors. We believe that an emphasis on histopathologic study is essential to confirm the clinical suspicion.

## Background

Oral focal mucinosis (OFM) is a rare disease; in 1974, Tomich [1] proposed that OFM was an oral manifestation of cutaneous focal mucinosis, which is characterized by focal myxoid degeneration of connective tissue. On clinical examination, it presents as a painless mass and has the same color as the surrounding mucosa [2]. While the etiology of OFM remains unknown, a possible cause is overproduction of hyaluronic acid by fibroblasts [3]. In this report, we summarize three cases of OFM occurring in the retromolar and gingival regions, in conjunction with a literature review. Occurrence in the retromolar region, as in Case 1 of our report, is rare.

## Case presentation

### Case 1

**Fig. 1:**
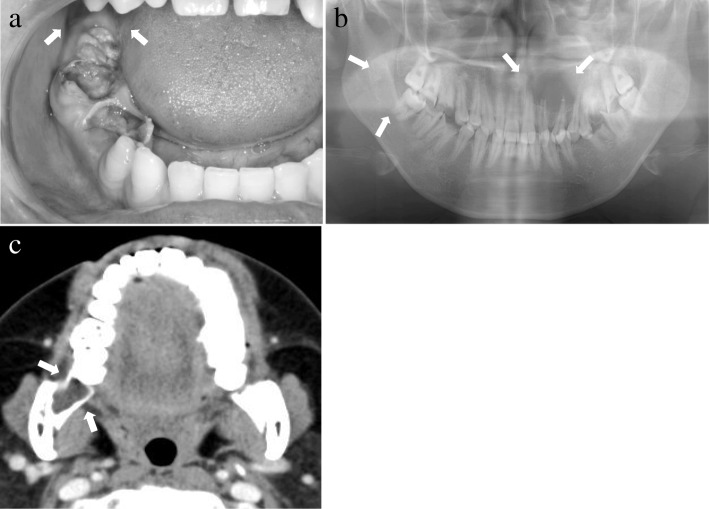
Diagnosis of Case 1 (photograph, radiograph, and computed tomography). **a** Intraoral photograph of Case 1 at the first visit. No obvious swelling was observed in the right retromolar region (arrow). **b** Panoramic radiograph of Case 1. Radiolucent lesions were observed in the left maxillary anterior tooth and right retromolar regions (arrow). **c** Contrast computed tomography image of Case 1. A clearly delineated low-density area depicted bone resorption in the right retromolar region, with no evidence of contrast effect (arrow)

A 26-year-old Japanese man was referred to our hospital in February 2008 with a chief complaint of swelling in the alveolar region of a maxillary anterior tooth, which had been present for the prior month. An intraoral examination revealed alveolar swelling on the labial side of the maxillary anterior tooth region. The mucosa of the retromolar region exhibited a normal color and no evident swelling (Fig. 1a). A panoramic radiographic examination revealed well-demarcated radiolucent lesions in the maxillary anterior tooth and the right retromolar regions (Fig. 1b). On computed tomography (CT), well-demarcated low-density areas, measuring 35 × 30 mm and 17 × 12 mm, were observed in the maxillary anterior tooth and right retromolar regions (Fig. 1c). The lesions were clinically diagnosed as a radicular cyst of the left lateral incisor and an additional suspected tumor of the right retromolar region. Pathological examination of the biopsy specimens revealed a radicular cyst of the left maxillary lateral incisor, and a suspected case of odontogenic myxoma in the right retromolar region. In May 2008, resection of the maxillary cyst and tumor of the retromolar region were performed under general anesthesia. The mucosa lining the retromolar region and the soft tissue of the bone defect were resected. No recurrence of either condition was observed at the final follow-up examination, 2 years later.

**Fig. 2:**
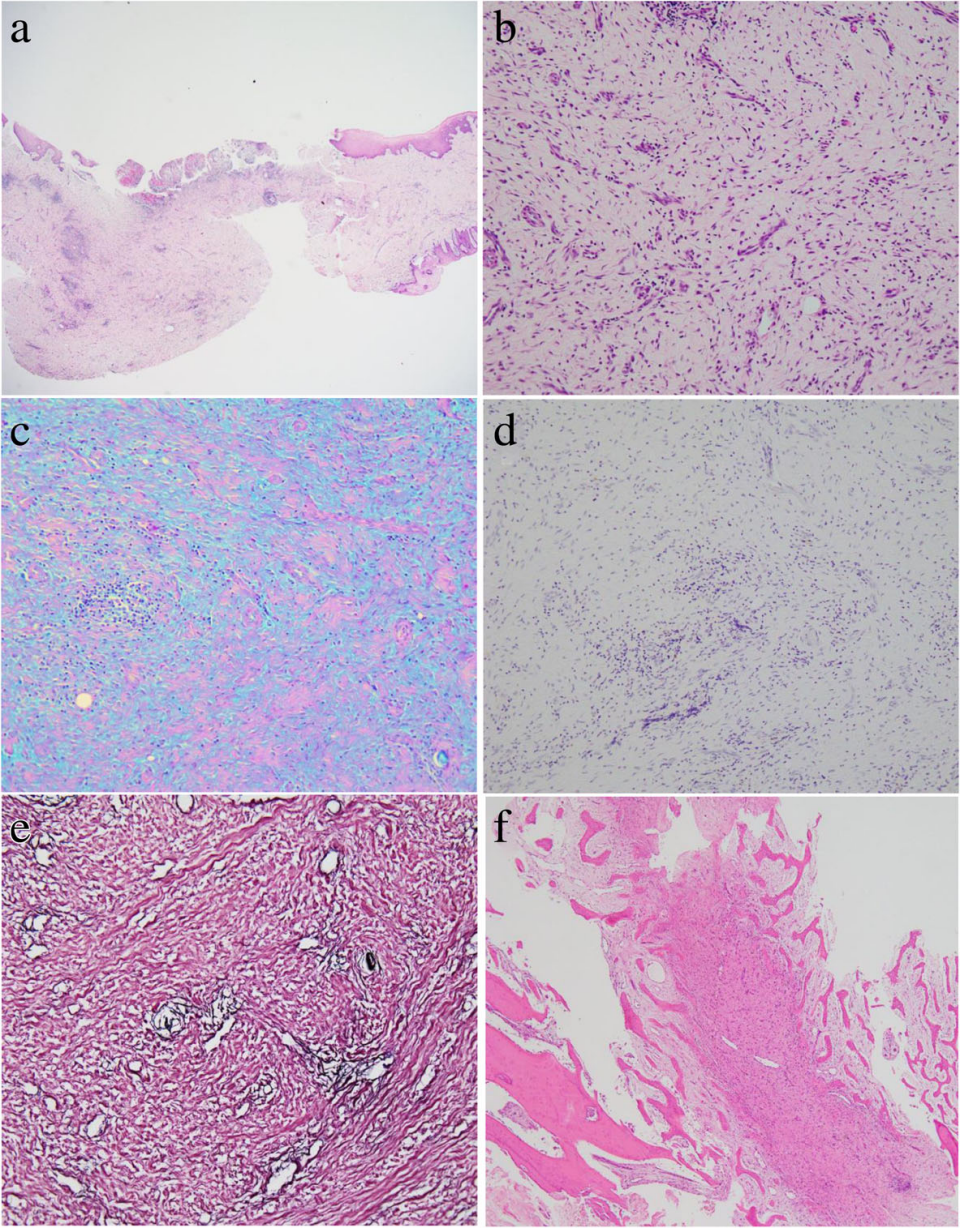
Histopathological images of Case 1. **a** Low-power magnification (hematoxylin and eosin stain, × 20 magnification). **b** High-power magnification, revealing a myxomatous stroma composition with sparse fibers (hematoxylin and eosin stain, × 200 magnification). **c** Myxomatous stroma exhibited Alcian blue stain positivity, and was negative for periodic acid–Schiff (Alcian blue periodic acid–Schiff stain, × 200 magnification). **d** Sparse formation of reticular fibers in the lesion (silver stain, × 200 magnification). **e** Negativity for S-100 protein (S-100 protein immunohistochemical stain, × 200 magnification). **f** Invasion of the surrounding bone was not observed (hematoxylin and eosin stain, × 40 magnification)

Histopathological examination identified stellate-shaped and spindle-shaped fibroblasts interspersed in an abundant myxoid matrix. Sparsely intercalated fibrous connective tissue was also observed (Fig. 2a, b). Alcian blue and periodic acid–Schiff (PAS) staining of the mucinous substrate of the tissue demonstrated a positive reaction with Alcian blue and a negative reaction with PAS (Fig. 2c). Sparse formation of reticular fibers was observed via the silver impregnation method (Fig. 2d). S-100 positive cells were not identified in immunohistochemistry (Fig. 2e). There was no clear encapsulation of the tissue mass, and an odontogenic epithelial island was also absent. Further, invasion of the peripheral bone by the tissue mass was not observed (Fig. 2f). A histopathological diagnosis of OFM was made.

### Case 2

**Fig. 3:**
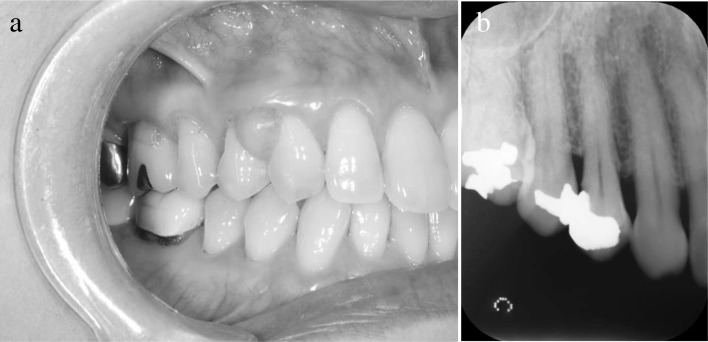
Diagnosis of Case 2 (photograph and radiograph). **a** Intraoral view of Case 2 at the initial visit. A 7 × 6-mm lesion was observed on the buccal surface of the maxillary right canine and first premolar region with healthy-colored mucosa. **b** Dental radiograph of Case 2. No obvious bone resorption was observed at the maxillary right canine and first premolar region

A 60-year-old Japanese woman visited our department in January 2015 with a chief complaint of a mass at the maxillary right canine and first premolar region, which had been identified during a visit to a private dental clinic in April 2014 for dental treatment and was still present at follow-up in January 2015. An intraoral examination revealed a 7 × 6-mm mass with elastic hardness and no mobility on the buccal gingiva at the maxillary right canine and first premolar region. The surface mucosa was a normal color, and the mass was painless and non-pedunculated (Fig. 3a). Dental radiographs did not show any obvious resorption of bone at the maxillary right canine and first premolar region (Fig. 3b). A clinical diagnosis of epulis of the gingiva was made. The mass was resected under local anesthesia in February 2015. No recurrence of the mass was observed at the final follow-up, 2 years after the surgical procedure.

**Fig. 4:**
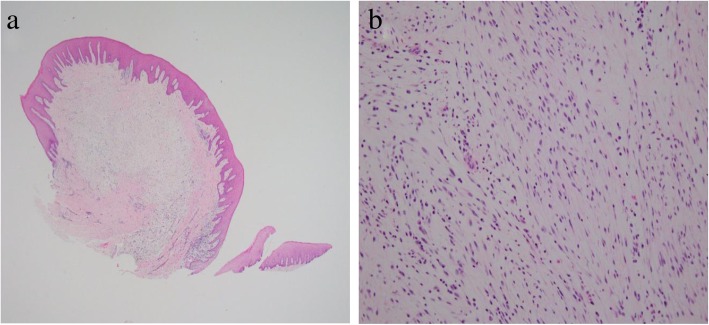
Histopathology of Case 2. **a** Low-power magnification (hematoxylin and eosin stain, × 20 magnification). **b** High-power magnification, showing a myxomatous stroma composition with a sparsity of fibers (hematoxylin and eosin stain, × 200 magnification)

Histopathological examination identified a myxomatous stroma with well-delineated borders and few fibers (Fig. 4a, b). The myxomatous stroma was positive for Alcian blue and negative for PAS. Silver staining did not identify the presence of any reticular fibers. S-100-positive cells were not observed. OFM was diagnosed based on the aforementioned findings.

### Case 3

**Fig. 5:**
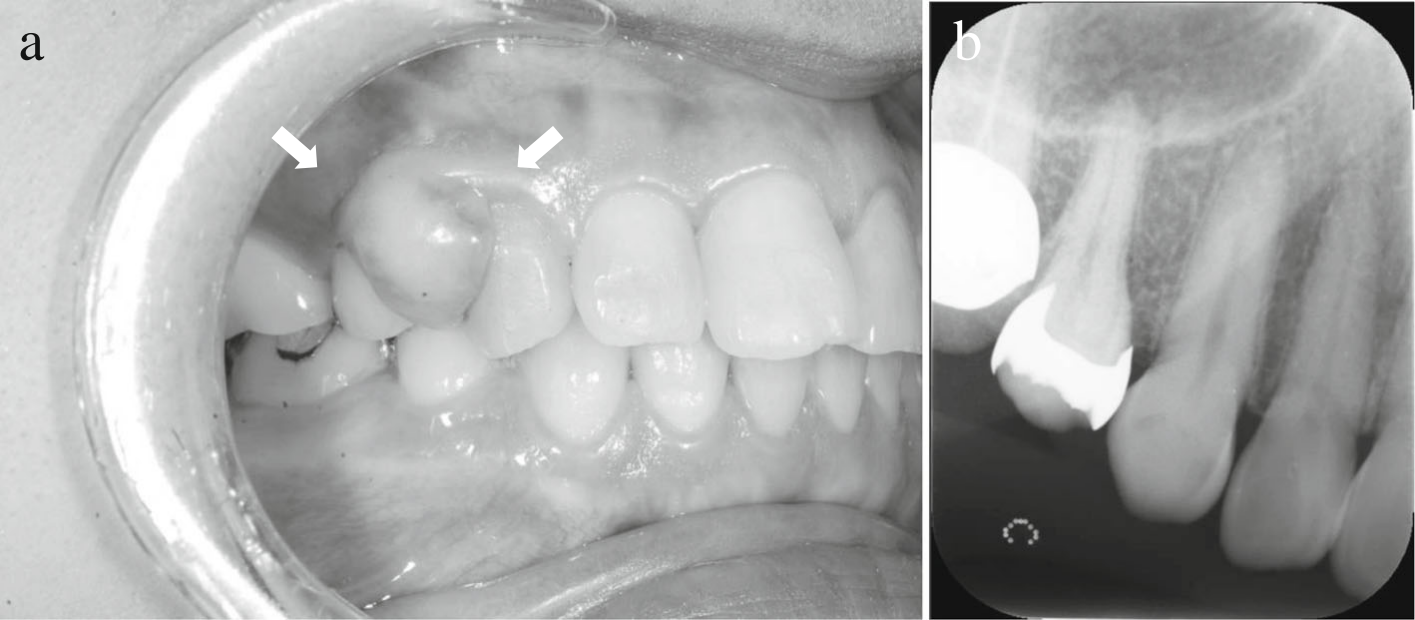
Diagnosis of Case 3 (photograph and radiograph). **a** Intraoral photograph of Case 3 at the initial visit. A 10 × 10-mm lesion was identified at the maxillary right canine and first premolar region of the buccal gingiva, with partial redness of the mucosa (arrow). **b** A dental radiograph of Case 3. There was no definitive bone resorption at the maxillary right canine and first premolar region

A 47-year-old Japanese woman presented to our department in October 2016 with a chief complaint of a mass on the buccal gingiva at the maxillary right canine and first premolar region, which she had been aware of since September 2015. An intraoral examination revealed a 10 × 10-mm mass with elastic hardness and no mobility on the buccal gingiva at the maxillary right canine and first premolar region. There was partial redness of the surface mucosa, and the mass was painless and non-pedunculated (Fig. 5a). No clear evidence of bone resorption at the maxillary right canine and first premolar region was observed on the dental radiograph (Fig. 5b). A clinical diagnosis of epulis of the gingiva was made. The mass was resected under local anesthesia in November 2016. No recurrence was observed at the final follow-up, 1 year after the surgical procedure.

**Fig. 6:**
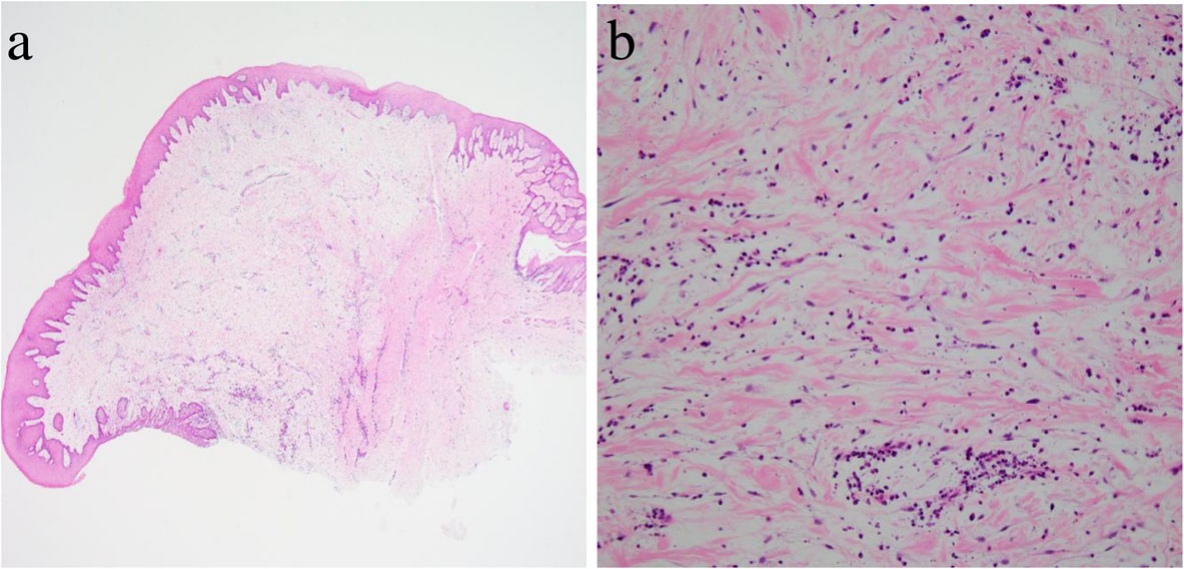
Histopathology of Case 3. **a** Low-power magnification (hematoxylin and eosin stain, × 20 magnification). **b** High-power magnification, showing myxomatous stroma with a sparsity of fibers and mild infiltration of plasma cells around the periphery of blood vessels (hematoxylin and eosin stain, × 200 magnification)

On histopathological examination, the gingival growth was well delineated with a myxomatous stroma characterized by a sparsity of fibers. There was mild infiltration of plasma cells around the periphery of the blood vessels (Fig. 6a, b). The myxomatous stroma was positive for Alcian blue and negative for PAS, but no reticular fibers were identified on silver staining. No S-100-positive cells were observed. A histopathological diagnosis of OFM was made.

## Discussion and conclusions

OFM is a rare mucosal disease of unknown etiology. While the pathophysiology of OFM is not clearly understood, it is characterized by the local deposition of mucin in connective tissue in which there has been mucoid degeneration [1]. To the best of our knowledge, there have been 65 reported cases of this disease in the English literature [1–23] (Table 1). The age of the patients ranged from 2 to 68 years, with a mean age of 38.4 years. Twenty-one cases involved male patients (32.3%), while 44 involved female patients (67.7%), yielding a male:female ratio of 1:2.1. Cases of gingival origin were the most prevalent (65.6%), followed by cases with origins in the hard palate, buccal mucosa, tongue, retromolar region, and lip.

**Table 1:** Classification of the 65 reported cases of oral focal mucinosis identified in the literature [1–23]

Classification	Frequency
Age (years)	Mean 38.4, range 2–68
Male:female ratio	21:44
Region of origin	
gingiva	44 (65.6%)^a^
palate	9 (13.4%)^b^
buccal mucosa	5 (7.5%)
tongue	4 (6.0%)
retromolar	3 (4.5%)^a^
lip	1 (1.5%)
unknown	1 (1.5%)
Clinical diagnosis	
fibroma	22 (32.8%)
epulis	7 (10.4%)
papilloma	2 (3.0%)
mucocele	2 (3.0%)
periodontal abscess	1 (1.5%)
giant cell granuloma	1 (1.5%)
pleomorphic adenoma	1 (1.5%)
benign tumor	1 (1.5%)
unknown	30 (44.8%)
Treatment method	
surgical resection	67 (100%)
Recurrence	1 (1.5%)

^a^Including the same diseases that presented in the gingiva and retromolar region

^b^Including the same diseases that presented on both sides of the palate

OFM is reportedly most likely to occur on keratinized mucosa attached to the bone [1]; cases of gingival and palatal origin together comprise 79.0% of the reported cases. Occurrence in the retromolar region, as in Case 1 of our report, is rare. The overproduction of hyaluronic acid by fibroblasts and consequent formation of myxoid lesion due to its accumulation has been hypothesized as a mechanism involved in this condition [1]. While the etiology is unknown, Neto *et al*. [15] have proposed traumatic stimulation as an eliciting factor in the disease mechanism. Moreover, Joshi *et al*. [18] suggested that traumatic stimulation may be involved in the increase in size of soft tissue lesions. However, we were unable to identify any obvious involvement of traumatic stimuli in the current cases.

The clinical findings of OFM include a painless nodular mass of elastic hardness with a similar color as that of the surrounding mucosa. However, there are no characteristic clinical and radiological features; accidental findings during dental treatment over a period of 10 years (from the first presentation of symptoms to diagnosis) have been reported [1, 4, 7].

The clinical diagnosis of OFM is particularly difficult, and the diagnoses in previous reports have included fibroma (32.8%), epulis (10.4%), papilloma (3.0%), mucocele (3.0%), benign tumor (1.5%), periodontal abscess (1.5%), and giant cell granuloma (1.5%), as well as pleomorphic adenomas in cases of lesions of palatal origin [23] (Table 1). A large number of cases (44.8%) were unidentified, with unrecorded clinical diagnoses at the first medical visit. None of these cases were diagnosed as OFM based on the clinical findings. Among the present cases, the first patient presented with no oral symptoms. Clearly delineated bone resorption in the right retromolar region was incidentally observed on the panoramic radiograph and diagnosed as a suspected retromolar tumor. Diagnoses of epulis were made in Cases 2 and 3, based on the presence of localized tissue masses on the buccal gingiva at the maxillary right canine and first premolar regions. Although it is difficult to tentatively diagnose OFM based on clinical symptoms and radiological findings, this disease should be considered in a differential diagnosis for benign oral tumors.

Pathological examination, including immunostaining, is essential for the definitive diagnosis of OFM. Histopathological findings include lack of encapsulation of the neoplastic tissue mass, a myxomatous stroma, and—in cases where a myxomatous stroma is absent—localized fibrous connective tissue [1]. Therefore, histopathological distinction from diseases with a myxomatous stroma, including myxoma, mucocele, nerve sheath myxoma, neurofibroma accompanied by mucus degeneration, focal myxedema, and mucoid degeneration of fibrotic lesions, is important [1].

In the present cases, Alcian blue and PAS staining revealed the deposition of a myxomatous substance between fibrous tissues. While positive for Alcian blue, the negative response for PAS was suggestive of the presence of acidic mucin. Furthermore, the possibility of mucoid degeneration of a peripheral nerve-derived lesion, such as nerve sheath myxoma, was eliminated based on the negative result for S-100 protein, which is an immunohistochemical marker of neural tissues or lesions. In addition, sparse fibrous connective tissue was observed in the present lesions, but reticular fibers were barely visible on silver staining.

In Case 1, bone resorption in the right retromolar region was observed, consistent with the tissue mass, and odontogenic myxoma was identified as a differential diagnosis based on the biopsy findings. However, a definitive diagnosis was not reached. Odontogenic myxoma is a true neoplasm of mesenchymal origin. It mainly consists of spindle-shaped cells and scattered collagen fibers distributed through a loose, mucoid material. Unlike OFM, odontogenic myxoma invariably presents as an intraosseous expansile lesion causing slow-growing enlargement of the jaw bone. Odontogenic myxoma and OFM are differentiated based on the arrangement and course of reticular fibers [3, 11]. Silver staining identified very sparse reticular fibers in this case. Hence, myxoma, which shows abundant reticular fiber formation, was ruled out. These findings pertaining to the present soft tissue mass were congruent with those described by Tomich [1], with an additional lack of invasion of the surrounding bone. There is no clear encapsulation in OFM, unlike tumors, and OFM lesions consist of a localized area of relatively thick myxomatous tissue surrounded by normal collagenous tissue; this histological feature is thought to be important in the differentiation of OFM and other diseases. Thus, a definitive diagnosis of OFM was established. However, in Case 1, resorption of the jaw bone, which is unusual for OFM, was also seen, which made an accurate clinical diagnosis difficult. There was only one case other than our case report that reported bone resorption in the radiological findings [23]. Even though radiographic findings have typically shown the mandibular cases of odontogenic myxoma to be multilocular [24], in these cases, the masses were unilocular on radiography; we presume that bone absorption was caused by compression with increasing size of the lesion.

Systemic diseases indicating mucinosis include pretibial myxedema occurring with hyperthyroidism, myxedema diffusum with hypothyroidism, scleredema and multiple myeloma due to diabetes, and lichen myxedematosus due to diabetes or collagenosis. In the present cases, these systemic diseases were not observed; in addition, because mucinosis was limited to the oral region, the possibility of systemic mucinosis was excluded. We believe it may be worthwhile to consider performing blood tests to eliminate endocrinological diseases after pathological diagnosis of OFM.

Thus far, treatment for all reported cases of OFM has been surgical resection. Of all the reported cases, there has been only one recurrence (1.5%) due to incomplete resection [16]; progress was satisfactory in the remaining reported cases. The current series showed no recurrence; however, a certain period of follow-up observation may be necessary in most cases.

Here we present three cases of OFM along with a review of the literature. Although OFM is difficult to diagnose on the basis of clinical findings and its frequency is low, the disease should be considered when diagnosing benign oral tumors.

## Abbreviations

CT: Computed tomography
OFM: Oral focal mucinosis
PAS: Periodic acid–Schiff
